# *QuickStats:* Infant Mortality Rates For Metro and Nonmetro Counties,* by Race and Hispanic Origin — National Vital Statistics System, United States, 2017

**DOI:** 10.15585/mmwr.mm6903a6

**Published:** 2020-01-24

**Figure:**
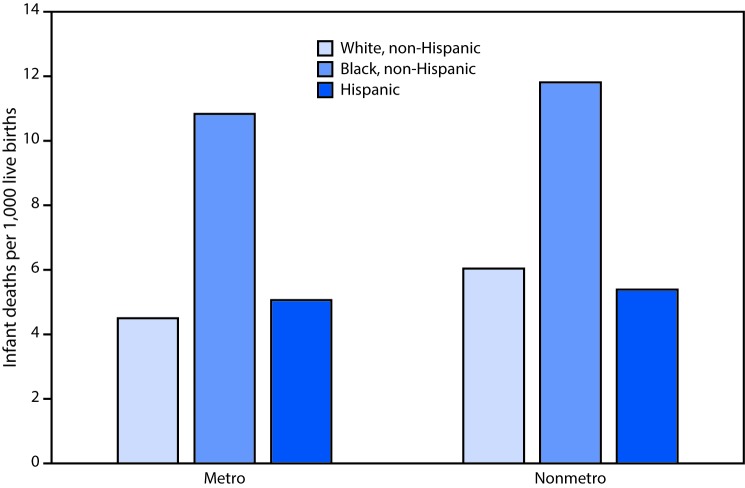
In metropolitan counties, infant mortality rates were lowest for infants of non-Hispanic white mothers (4.50 infant deaths per 1,000 live births), followed by rates for infants of Hispanic mothers (5.08) and highest for infants of non-Hispanic black mothers (10.84). In nonmetropolitan counties, the mortality rate was lowest for infants of Hispanic mothers (5.38) followed by infants of non-Hispanic white mothers (6.05) and highest for infants of non-Hispanic black mothers (11.81). The infant mortality rate was significantly lower for infants of non-Hispanic white women in metro counties compared with nonmetro counties; differences in rates between metro and nonmetro counties for the two other groups were not significant.

**Source:** National Vital Statistics System. Linked birth/infant death period file, 2017.

